# Outpatient Follow-Up Visits to Reduce 30-Day All-Cause Readmissions for Heart Failure, COPD, Myocardial Infarction, and Stroke: A Systematic Review and Meta-Analysis

**DOI:** 10.5888/pcd21.240138

**Published:** 2024-09-26

**Authors:** Dylan J. Bilicki, Mathew J. Reeves

**Affiliations:** 1 College of Human Medicine, Michigan State University, East Lansing; 2 Department of Epidemiology and Biostatistics, Michigan State University, East Lansing

## Abstract

**Introduction:**

Hospital readmissions is an important public health problem that US hospitals are responsible for reducing. One strategy for preventing readmissions is to schedule an outpatient follow-up visit before discharge. The objective of this study was to determine whether outpatient follow-up visits are an effective method to reduce 30-day all-cause readmissions for patients discharged from US hospitals with heart failure, chronic obstructive pulmonary disease (COPD), acute myocardial infarction (AMI), or stroke.

**Methods:**

We conducted a systematic review and meta-analysis to identify relevant articles published from 2013 through 2023. We searched PubMed, CINAHL, and Cochrane. Eligible studies were those that assessed the effect of postdischarge outpatient follow-up visits on 30-day all-cause readmission. We used random effect meta-analyses to generate pooled adjusted effect estimates and 95% CIs.

**Results:**

We initially identified 2,256 articles. Of these, 32 articles underwent full-text review and 15 met inclusion criteria. Seven studies addressed heart failure, 3 COPD, 2 AMI, and 3 stroke. Ten articles provided sufficient information for meta-analysis. The pooled adjusted effect measure was 0.79 (95% CI, 0.69–0.91), indicating that outpatient follow-up visits were associated with a 21% lower risk of readmission. However, we found a high degree of between-study heterogeneity (*Q* = 122.78; *P* < .001; *I* ^2^ = 92.7%). Subgroup analyses indicated that study quality, disease condition, and particularly whether a time-dependent analysis method was used, explained much of the heterogeneity.

**Conclusion:**

Outpatient follow-up visits are a potentially effective way to reduce 30-day all-cause readmissions for patients discharged with heart failure or stroke, but evidence of benefit was lacking for COPD and we found no studies for assessing AMI. Our results emphasize the importance of study quality.

**Summary**

**What is already known on this topic?**

Outpatient follow-up visits soon after discharge may help prevent hospital readmissions.

**What is added by this report?**

The pooled adjusted effect of outpatient follow-up visits reduced 30-day all-cause readmissions by 21%, but between-study variability was high (*I* ^2^ = 92.7%).

**What are the implications for public health practice?**

Health care systems should continue to encourage the scheduling of outpatient follow-up visits, but more high-quality research studies are needed.

## Introduction

Hospital readmissions are a serious public health problem and are associated with increased illness, death, and health care costs (1). An estimated 3.8 million readmissions occurred in the US in 2018 with an average cost of $15,200 per readmission (1,2). Heart failure, chronic obstructive pulmonary disease (COPD), acute myocardial infarction (AMI), and stroke are 4 highly prevalent conditions in the top 20 leading causes of readmissions. In 2018, some 1 million index heart failure admissions resulted in 233,000 readmissions and cost $3.49 billion (1). Similarly, in that year, COPD, AMI, and stroke accounted for 78,000, 74,300, and 53,000 readmissions, respectively, with readmission rates ranging from 10% to 20% (1,2). Patients who are readmitted also have poorer outcomes, including lower survival rates and poorer quality of life (3–5), when compared with patients discharged with the same disease who are not readmitted.

In 2013, the Hospital Readmissions Reduction Program (HRRP) began offering incentives to hospitals with low readmission rates and enforcing penalties on hospitals with high readmission rates (6,7). The HRRP targets heart failure, COPD, and AMI. Stroke was proposed for inclusion, but controversy over the importance of stroke severity led to its exclusion (8). Controversy remains regarding the effectiveness of HRRP in reducing readmissions (9,10).

Studies on transitional care services aimed at reducing readmissions showed promising results (11–13), but uncertainty about their effectiveness remains (14–16), in part due to barriers such as insufficient administrative support, lack of resources, and lack of staff buy-in (17). A previous meta-analysis of randomized trials that focused on reducing heart failure readmissions included various interventions, such as patient education, telephone support, nurse home visits, and outpatient follow-up visits (11). The meta-analysis concluded that nurse home visits and outpatient follow-up visits were effective in reducing readmissions, but because each trial tested at least 2 interventions bundled together, it was difficult to isolate the effect of any single strategy. The objective of this study was to quantify the singular effect of outpatient follow-up visits on reducing 30-day all-cause readmissions in patients with heart failure, COPD, AMI, or stroke discharged from US acute care hospitals from 2013 through 2023.

## Methods

We conducted this systematic review and meta-analysis according to the Preferred Reporting Items for Systematic Reviews and Meta-Analysis (PRISMA) (18). Briefly, we searched 3 databases (PubMed, CINAHL, and Cochrane) by using a combination of terms that included but was not limited to heart failure, COPD, AMI, stroke, readmission, rehospitalization, outpatient, office, follow-up, post discharge, and visit. The search was completed on June 14, 2023, and included all studies published on or after January 1, 2013 (ie, approximately 10.5 years). Both authors independently screened the titles and abstracts of the initial list of citations, identifying potentially eligible articles for full-text review. We conducted an additional review of the bibliographies of 7 related meta-analyses identified by our search. We resolved disagreements on initial and final study selection by consensus.

### Study selection

Eligible studies were those that 1) included patients aged 18 years or older, who were discharged from US hospitals with an index hospitalization for heart failure, COPD, AMI, or stroke, 2) identified the presence or absence of an outpatient follow-up visit within 30 days of discharge as the primary exposure variable, 3) used 30-day all-cause readmission as the primary outcome, and 4) studied either the direct effect of receiving an outpatient follow-up visit within 30 days of discharge or assessed the effect of scheduling an appointment for an outpatient follow-up visit before discharge. We limited outpatient follow-up visits to those occurring in a traditional ambulatory setting with either a primary care or specialist physician, physician’s assistant, or nurse practitioner. We included all types of study designs, including retrospective cohorts, case-control studies, clinical trials, and quality improvement projects that used a pre–post comparative design. We did not include studies that assessed outpatient follow-up visits that had to occur at 1 specific outpatient clinic (eg, a clinic at the discharging hospital) or those that examined the effect of outpatient follow-up visits that only involved a pharmacist. However, we included studies that included pharmacists as part of a multidisciplinary team. We also excluded studies with sample sizes less than 100, editorials, and abstracts.

### Data extraction

For studies that underwent full-text review and met all eligibility criteria, we extracted data on the following study-level characteristics: study design, condition or diagnosis, geographic location (ie, city, state, region), objective of the study, data source (electronic medical record, administrative data, disease registry), sample size, time frame of case enrollment, discharge destinations (various combinations of home, home health, skilled nursing facility, acute rehabilitation, hospice, other), description of exposure (type of provider, timing postdischarge), outcome (30-day readmission), whether the analysis was conducted at the patient or hospital level, prevalence of outpatient follow-up, crude readmission rate, adjusted effect measure (either an odds ratio [OR] or hazard ratio [HR]), 95% CIs, and *P* values. Data were extracted in duplicate by both authors, and differences were resolved by consensus.

To assess study quality, we modified the Newcastle-Ottawa Scale, which assesses the quality of nonrandomized studies (19). We made 2 modifications: we assessed whether the study adequately controlled for demographic variables (age, race, sex, socioeconomic status), and we added a new item referred to as “time-dependent bias.” We added this item to address a common problem associated with readmission studies (20), whereby subjects who have a readmission soon after discharge do not have the opportunity to have an outpatient follow-up visit, so they remain “unexposed.” Our modified scale had 8 binary (yes or no) quality criteria and a total score ranging from 0 to 8. We used 3 criteria (representativeness of exposed cohort [whether the study population was broadly representative of the US population in terms of age, sex, ethnicity, and socioeconomic status], selection of nonexposed cohort, and ascertainment of exposure) to assess selection of study participants, 3 criteria (control for demographics, control for severity of disease or readmission risk, and time-dependent bias) to assess comparability of exposure groups, and 2 criteria (assessment of outcome and adequacy of follow-up of cohorts) to assess outcomes. We used scores of less than 6 to define low-quality studies.

We generated descriptive statistics to describe the characteristics of the included studies. For the studies that provided an adjusted effect measure (OR or HR) that quantified the effect of outpatient follow-up visits on 30-day readmission risk at the patient level, we conducted a random-effect (DerSimonian–Laird) meta-analysis using the meta command in Stata version 16 (StataCorp LLC). We categorized these reports as Tier 1 studies. We combined individual adjusted ORs or HRs without further manipulation to create a pooled adjusted effect estimate (labeled OR/HR), and calculated 95% CIs. We used the Cochrane *Q *statistic to test for between-study heterogeneity and the *I* ^2 ^statistic to quantify the magnitude of between-study heterogeneity. A *Q* statistic with an associated *P* value less than .05 indicates a significant amount of between-study heterogeneity. An *I* ^2 ^statistic greater than 30% indicates a moderate degree of between-study heterogeneity, and an *I* ^2 ^statistic greater than 75% indicates a high degree of heterogeneity. Prespecified subgroup analyses included study quality (score of ≥6 [high] vs <6 [low]), adequate control of time-dependent bias (controlled or not controlled), and diagnosis (heart failure, COPD, AMI, stroke). We conducted these subgroup analyses to determine whether these study-level characteristics influenced the effect of outpatient follow-up visits in reducing the risk of 30-day all-cause readmission. Quality improvement projects did not provide an adjusted effect measure for outpatient follow-up visits and were, therefore, not included in the meta-analysis. Similarly, comparative studies that presented results aggregated at the hospital level rather than at the patient level were also not included in the meta-analysis. We categorized these 2 types of reports as Tier 2 studies and reviewed them qualitatively.

## Results

**Figure 1:**
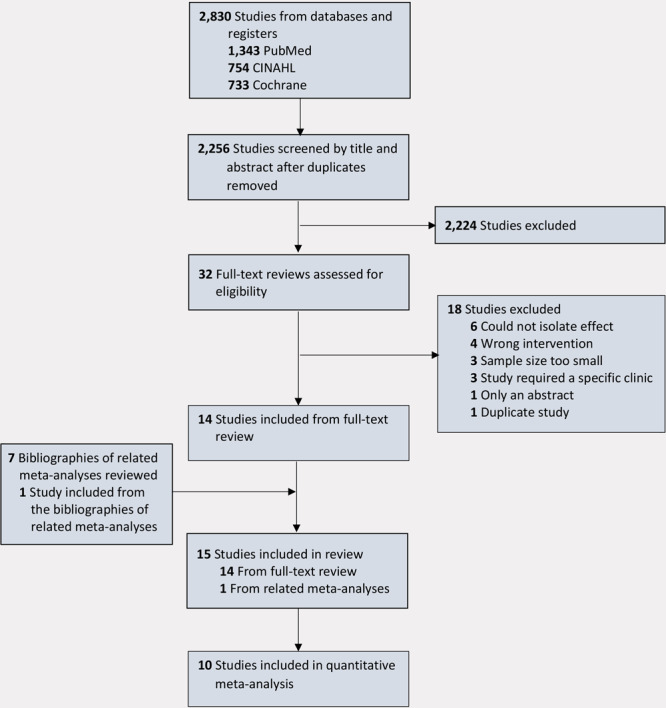
PRISMA (Preferred Reporting Items for Systematic Reviews and Meta-Analysis) flow diagram of systematic review of US studies of outpatient follow-up visits and reduction of 30-day all-cause readmissions among patients with heart failure, chronic obstructive pulmonary disease, acute myocardial infarction, or stroke. Abbreviation: CINAHL, Cumulative Index to Nursing and Allied Health Literature.

Our search of the 3 databases yielded 2,830 citations, which after removing 574 duplicates yielded 2,256 unique citations (Figure 1). After applying exclusion criteria, 32 studies underwent full-text review, and 15 articles were included in our final review (Table 1); 10 articles were Tier 1 studies, and 5 articles were Tier 2 studies. 

**Table 1:** Systematic Review of US Studies of Outpatient Follow-Up Visits and Reduction of 30-Day All-Cause Readmissions Among Patients With Heart Failure, COPD, Acute Myocardial Infarction, or Stroke: January 1, 2013–June 14, 2023

Study, date of publication	Disease	Sample size	Study design	Exposure	Location (study period)	Data source	Discharge destination
**Tier 1 studies^a^**							
Fidahussein et al (21), 2014	COPD	839	Retrospective cohort	Visit with a PCP or pulmonologist within 30 days of discharge	Olmsted County, Minnesota (2004–2011)	Mayo Clinic EMR	Home; skilled nursing facility
Sharif et al (22), 2014	COPD	8,263	Retrospective cohort	Visit with a PCP, pulmonologist, or both within 30 days of discharge	US (2009–2011)	Optum Insight, a national claims database	Anywhere but long-term care health center
Budde et al (23), 2019	COPD	2,653	Retrospective cohort	Visit with a PCP within 10 days of discharge	New York City (2011–2016)	Mount Sinai Hospital EMR	Anywhere but hospice
Lee et al (24), 2016	Heart failure	11,985	Case control study	Visit with a PCP or cardiologist within 7 days of discharge	Northern California (2006–2013)	Kaiser Permanente EMR	Home
Baecker et al (25), 2020	Heart failure	26,128	Retrospective cohort	Visit with a PCP or nurse practitioner within 7 days of discharge	Southern California (2013–2018)	Kaiser Permanente EMR	Home; home health care
Distelhorst and Hansen (26), 2022	Heart failure	1,280	Retrospective cohort	Visit with a PCP within 14 days of discharge	Ohio (2017–2019)	Cleveland Clinic Health System EMR	Home
Xu et al (27), 2022	Heart failure	6,918	Retrospective cohort	Visit with a PCP, geriatrician, or cardiologist within 14 days of discharge	Duke University Health System, North Carolina (2020–2021)	Duke University Health System EMR	Home; home health care
Terman et al (28), 2018	Stroke	78,345	Retrospective cohort	Visit with a PCP or neurologist within 30 days of discharge	US (2012)	Fee-for-service Medicare claims	Home
Leppert et al (29), 2020	Stroke	14,630	Retrospective cohort	Visit with a PCP or neurologist within 30 days of discharge	US (2009–2015)	PharMetrics, a national claims database	Home
Hussein et al (30), 2022	Stroke	872	Retrospective cohort	Visit with a PCP within 30 days of discharge prestroke and poststroke	Twin Cities, Minnesota (2015–2018)	University of Minnesota hospital EMR	Home; home health care; skilled nursing facility; long-term care health center
**Tier 2 studies^b^**							
Hess et al (31), 2013	Acute myocardial infarction	228 hospitals	Retrospective cohort	Visit with any physician within 7 days of discharge, measured at the hospital level	US (2003–2006)	CRUSADE registry data linked to Medicare fee-for-service claims	Home
Brown et al (32), 2014	Acute myocardial infarction	1,088 hospitals	Retrospective cohort	Visit with a PCP within 14 days of discharge, measured at the hospital level	US (2008–2009)	MedPAR, a national database	Home; home health care
Ryan et al (33), 2013	Heart failure	398 patients, 1 hospital	Quality improvement project	Visit with a cardiologist within 7 days of discharge, patients identified as preintervention or postintervention	Connecticut (2008–2011)	Fee-for-service Medicare claims	Not reported
Baker et al (34), 2015	Heart failure	56,072 patients, 20 hospitals	Quality improvement project	Visit with any physician within 7 days of discharge, patients identified as preintervention or postintervention	Southeastern Michigan (2011–2013	Fee-for-service Medicare claims	Home
Dev et al (35), 2021	Heart failure	261 patients, 1 hospital	Quality improvement project	Visit with a cardiologist within 7 to 14 days of discharge, patients identified as preintervention or postintervention	Phoenix, Arizona (2010–2013)	Phoenix Veterans’ Administration Medical Center EMR	Home; home health care

Abbreviations: COPD, chronic obstructive pulmonary disease; CRUSADE, Can Rapid Risk Stratification of Unstable Angina Patients Suppress Adverse Outcomes with Early Implementation of the ACC/AHA Guidelines; EMR, electronic medical record; MedPAR, Medicare Provider Analysis and Review; PCP, primary care physician.

a Studies that provided an adjusted effect measure (odds ratio or hazard ratio) that quantified the effect of outpatient follow-up visits on 30-day readmission risk at the patient level were categorized as Tier 1 studies.

b Quality improvement projects that did not provide an adjusted effect measure for outpatient follow-up visits and comparative studies and presented results aggregated at the hospital level rather than at the patient level were categorized as Tier 2 studies.

### Tier 1 studies

Nine of 10 Tier 1 studies used a retrospective cohort design; one used a case-control design (Table 1). Tier 1 studies had a wide range of sample sizes, from 839 to 78,345 participants. Every Tier 1 study defined the exposure as an outpatient follow-up visit with a primary care physician or a specialist physician (cardiologist, pulmonologist, geriatrician, neurologist), or a nurse practitioner within 30 days of discharge. We found significant differences in geographic location. Three studies used national data (either large claims-based or fee-for-service Medicare data); the remaining 7 studies used electronic medical records from health systems of various sizes (range, 1 to 26 hospitals). We also found differences in the combination of hospital discharge destinations used to select eligible participants. Every study included home with or without home health as a discharge destination, but varied in whether they included other destinations such as skilled nursing facilities or long-term care hospitals.

### Meta analysis

**Figure 2:**
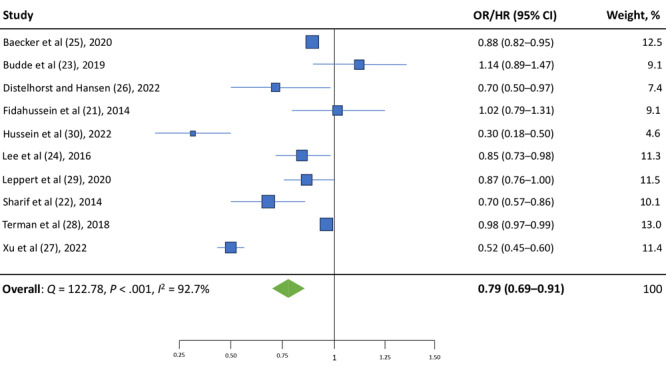
Random effect meta-analysis showing the pooled summary estimate of all 10 Tier 1 studies. The size of the data markers (squares) corresponds to the weight of the study in the meta-analysis. Abbreviations: HR, hazard ratio; OR, odds ratio.

The random effects meta-analysis conducted on the 10 Tier 1 studies (Figure 2) found a significant overall pooled adjusted relative effect (OR/HR = 0.79; 95% CI, 0.69–0.91). However, we found a high degree of between-study heterogeneity (*Q* = 122.78; *P* < .001; *I* ^2^ = 92.7%). 

**Table:** 

Study	OR/HR (95% CI)	Weight, %
Baecker et al (25), 2020	0.88 (0.82–0.95)	12.5
Budde et al (23), 2019	1.14 (0.89–1.47)	9.1
Distelhorst and Hansen (26), 2022	0.70 (0.50–0.97)	7.4
Fidahussein et al (21), 2014	1.02 (0.79–1.31)	9.1
Hussein et al (30), 2022	0.30 (0.18–0.50)	4.6
Lee et al (24), 2016	0.85 (0.73–0.98)	11.3
Leppert et al (29), 2020	0.87 (0.76–1.00)	11.5
Sharif et al (22), 2014	0.70 (0.57–0.86)	10.1
Terman et al (28), 2018	0.98 (0.97–0.99)	13.0
Xu et al (27), 2022	0.52 (0.45–0.60)	11.4
Overall: I-squared = 92.7%; *P* <.001	0.79 (0.69–0.91)	100

### Quality assessment

Total scores for the 10 Tier 1 studies ranged from 4 to 8, with a median of 6 (Table 2). Three studies (22,23,30) were deemed low-quality (score <6). Studies scored poorly on 2 criteria in particular: representativeness of the exposed cohort and time-dependent bias. Only 2 studies (24,25) scored well in representativeness, and both were conducted in California using Kaiser Permanente health system data. The 3 studies (22,28,29) that used national databases did not have proportions of age, sex, ethnicity, and socioeconomic status close enough to the national averages to warrant a positive score in representativeness.

**Table 2:** Results of Application of Modified Newcastle-Ottawa Scale for Assessing the Quality of Nonrandomized Studies in a Systematic Review of Published US Studies of Outpatient Follow-Up Visits and Reduction of 30-Day All-Cause Readmissions Among Patients With Heart Failure, COPD, Acute Myocardial Infarction, or Stroke^a^: January 1, 2013–June 14, 2023

Study (date of publication)	Selection of study population			Comparability between exposure groups, control of confounding			Outcome		Score^b^
	Representativeness of exposed cohort	Selection of non-exposed cohort	Ascertainment of exposure	Control for demographic characteristics	Control for severity of disease or readmission risk	Time-dependent bias	Assessment of outcome	Adequacy of follow-up of cohorts	
Fidahussein et al (21), 2014		◆	◆	◆	◆		◆	◆	6
Sharif et al (22), 2014		◆	◆		◆		◆	◆	5
Budde et al (23), 2019		◆		◆	◆			◆	4
Lee et al (24), 2016	◆	◆	◆	◆	◆	◆	◆	◆	8
Baecker et al (25), 2020	◆	◆	◆	◆	◆	◆	◆	◆	8
Distelhorst and Hansen (26), 2022		◆	◆	◆	◆		◆	◆	6
Xu et al (27), 2022		◆	◆	◆	◆		◆	◆	6
Terman et al (28), 2018		◆	◆	◆	◆	◆	◆	◆	7
Leppert et al (29), 2020		◆	◆	◆	◆	◆	◆	◆	7
Hussein et al (30), 2022		◆	◆	◆			◆	◆	5

Abbreviation: ◆, study included this element.

a Only articles included in the meta-analysis (Tier 1 studies) were assessed for quality. Studies that provided an adjusted effect measure (odds ratio or hazard ratio) that quantified the effect of outpatient follow-up visits on 30-day readmission risk at the patient level were categorized as Tier 1 studies.

b Modified scale had 8 binary quality criteria and a total score ranging from 0 to 8. A score of <6 was considered low quality; a score of ≥6 was considered high quality.

Only 4 studies scored well on addressing time-dependent bias by using a method to ensure that the exposure (outpatient follow-up visit) occurred before the outcome (readmission). One study (24) did this at the study design phase by individually matching cases and controls on the duration of follow-up time available. The other 3 studies (25,28,29) controlled time-dependent bias at the analysis stage by defining the exposure as a time-dependent variable in a Cox regression model.

### Subgroup analyses

**Figure 3:**
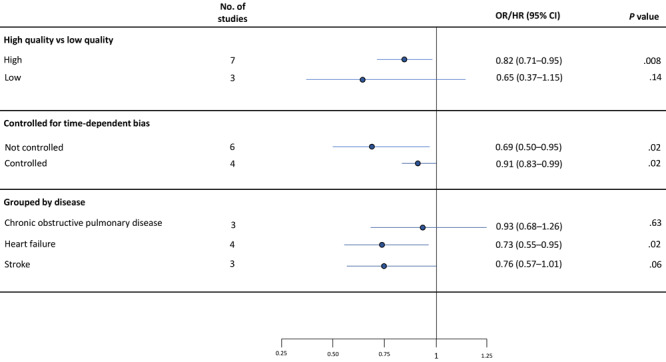
Random effect meta-analysis showing the pooled effect estimates for 3 subgroups.

The pooled adjusted effect of outpatient follow-up visits was smaller in the 7 high-quality studies (OR/HR = 0.82; 95% CI, 0.71–0.95; *P* = .008) than in the 3 low-quality studies (OR/HR = 0.65; 95% CI, 0.37–1.15; *P* = .14), although only the former was significant (Figure 3). Both subgroups showed high levels of between-study heterogeneity (high quality: *Q* = 91.49, *P* <.01, *I* ^2^ = 93.44%; low quality: *Q* = 22.82, *P* <.01, *I* ^2^ = 91.23%).

**Table:** 

Comparison	No of studies	OR/HR (95% CI)	*P* value
**High quality vs low quality**			
High quality	7	0.82 (0.71–0.95)	.008
Low quality	3	0.65 (0.37–1.15)	.14
**Controlled for time-dependent bias**			
Not controlled	6	0.69 (0.50–0.95)	.02
Controlled	4	0.91 (0.83–0.99)	.02
**Grouped by disease**			
Chronic obstructive pulmonary disease	3	0.93 (0.68–1.26)	.62
Heart failure	4	0.73 (0.55–0.95)	.02
Stroke	3	0.76 (0.57–1.01)	.06
**Overall**	—	0.79 (0.69–0.91)	.001

The 4 studies that adequately controlled for time-dependent bias demonstrated a smaller pooled effect and narrower 95% CIs (OR/HR = 0.91; 95% CI, 0.83–0.99; *P* = .03) than the 6 studies that did not control for this bias (OR/HR = 0.69; 95% CI, 0.50–0.95; *P* = .02) (Figure). Both subgroups demonstrated high levels of between-study heterogeneity (not controlled for bias: *Q* = 49.32, *P* <.01, *I* ^2^ = 89.86%; controlled for bias: *Q* = 14.11, *P* <.01, *I* ^2^ = 78.74%).

When we grouped studies by disease condition, the 4 heart failure studies showed a significant 27% reduction in readmission risk (OR/HR = 0.73; 95% CI, 0.55–0.95; *P* = .02) (Figure). The pooled adjusted effect among the 3 stroke studies was similar in magnitude but the confidence interval slightly exceeded the null value (OR/HR = 0.76; 95% CI, 0.57–1.01; *P* = .06). The pooled adjusted effect of the 3 COPD studies was smaller and not significant (OR/HR = 0.93; 95% CI, 0.68–1.26; *P* = .62). All 3 subgroups still showed high levels of between-study heterogeneity (heart failure: *Q* = 41.97, *P* < .01, *I* ^2^ = 92.85%; stroke: *Q* = 22.70, *P* < .01, *I* ^2^ = 91.19%; COPD: *Q* = 10.26, *P* = .01, *I* ^2^ = 80.51%).

### Description of Tier 2 studies

Five Tier 2 studies were not included in the meta-analysis. Two were retrospective cohort designs that included only AMI patients; both presented aggregated hospital-level data stratified by outpatient follow-up rates divided into quartiles. One (31) used Medicare claims data from 288 hospitals from a national registry and found that hospitals in the highest quartile for outpatient follow-up rates had similar readmission risk compared with the bottom quartile (OR = 0.99). The other retrospective cohort study (32) used national Medicare claims data from 1,088 hospitals and found that hospitals in the lowest quartile for outpatient follow-up rates had slightly higher risk of readmission (RR = 1.07) compared with the highest quartile of hospitals.

The 3 remaining studies (33–35) were quality improvement projects that used a pre–post design to measure the effectiveness of enhanced discharge planning strategies on increasing outpatient follow-up visits and decreasing 30-day all-cause readmissions in heart failure patients. All 3 projects were conducted at the regional or state level and had sample sizes ranging from 261 (35) to 56,072 patients (34). Two reports (33,35) were single-center studies, and the third (34) included 20 Michigan hospitals. The quality improvement interventions increased the frequency of outpatient follow-up visits from as low as 3.3% (34) to as high as 27.3% (33). The net impact of these studies on readmission risk showed similarly wide variation; one study (34) found only a modest decline of readmissions (1.7%), although because of the large study size this effect was significant. Dev et al (35) found a 9% decrease in readmissions postintervention, and Ryan et al (33) observed the largest decline in readmission risk (30%).

## Discussion

This systematic review included 15 US-based studies published since 2013 that reported on the effect of outpatient follow-up visits on the risk of 30-day all-cause readmission for heart failure, COPD, AMI, and stroke patients. The overall results of the meta-analysis that included 10 of these studies indicated a modest but significant 21% reduction in risk of readmission when heart failure, COPD, and stroke patients had an outpatient follow-up visit shortly after hospital discharge. However, when the effect of outpatient follow-up visits was stratified by disease condition, we observed a significant reduction in readmission only for heart failure and stroke. We found a relative risk reduction of 27% in heart failure patients that was similar in magnitude to another meta-analysis of heart failure patients that found a 20% reduction in readmission risk (RR = 0.80; 95% CI, 0.67–0.97) with the use of multiple interventions that included outpatient follow-up visits (11). We observed a similar risk reduction in stroke patients (24%), but we are not aware of any other meta-analyses conducted among stroke patients that report on the efficacy of outpatient follow-up visits in reducing readmission risk. Our systematic review did not yield any Tier 1 studies conducted among AMI patients; thus, AMI was not included in the meta-analysis. We hypothesize that the lack of studies examining outpatient follow-up visits among AMI patients may be due to the greater focus on cardiac rehabilitation in this population.

The estimated risk reduction in COPD patients who received an outpatient follow-up visit (7%) was noticeably smaller in magnitude than the estimated risk reduction for heart failure and stroke patients. The effect was also smaller than a previous meta-analysis that reported a 20% reduction in readmission risk for COPD patients exposed to bundled discharge interventions that included outpatient follow-up visits (RR = 0.80; 95% CI, 0.65–0.99) (36). However, we believe that the lack of an effect for COPD patients observed in our meta-analysis is best explained by the poor quality of the 3 COPD studies, none of which controlled for time-dependent bias (21–23).

Time-dependent bias (20,31) (also known as “survival bias” [20] or “immortal-time bias” [37,38]) occurs in studies that assess the effect of an exposure on an outcome when the classification of exposed participants requires that the participant remain event-free until they are exposed (20). Thus, in readmission studies, patients who are readmitted shortly after discharge may not have had an opportunity to complete their scheduled outpatient follow-up visit (and to become “exposed”), and therefore remain “unexposed,” resulting in a bias where the readmission rate is inflated in the group that did not have an outpatient follow-up visit. Time-dependent bias is common in observational studies (38,39) and is important to control for because the highest readmission rates observed in patients with COPD occurs in the first 72 hours after hospital discharge (40), which is likely to occur before an outpatient follow-up visit can be completed. A study by Zhou (20) and colleagues compared 5 methods of controlling for time-dependent bias and concluded that “exposure time matching” implemented during the design phase or defining the exposure as a “time-dependent variable” in the statistical analysis phase were the 2 best ways to control for time-dependent bias (20). These authors also found that ignoring the bias could almost double the effect estimate of the exposure (HR = 0.62 for no control vs HR = 0.80 when either of the above 2 methods were used). In our study, of the 4 Tier 1 articles that controlled for time-dependent bias, one (24) used the exposure-time–matching method during the design phase, and the other 3 (25,28,29) used a time-dependent variable in their statistical model. We observed similar findings to Zhou (20) and colleagues: our subgroup analysis showed that articles that ignored time-dependent bias estimated a 31% reduction in readmission risk, while the 4 articles that controlled time-dependent bias demonstrated only a 9% reduction in risk and a much narrower 95% CI.

Outpatient follow-up visits represent an important opportunity for hospitals and providers to prevent readmissions and improve patient outcomes (41), especially for heart failure and stroke patients. Scheduling outpatient follow-up visits at the time of discharge is a logical intervention for hospitals to use to reduce the risk of readmission for patients. However, while simple in theory, its implementation is often complicated when navigating the US health care system. Challenges related to lack of insurance, lack of a regular health care provider, costs, health literacy, and travel are just a few of the many barriers to implementing outpatient follow-up visits effectively (16,42). Beyond reducing readmissions, outpatient follow-up visits can present an opportunity for reconciling medications, building self-management skills, and ordering further medical testing (43). While outpatient follow-up visits show promising results, it is unlikely that a single intervention can fix the problems of readmissions on its own. Many studies have included outpatient follow-up visits as a part of a comprehensive set of interventions designed to reduce readmission risk (12–14,44,45), which have also been a focus of some meta-analyses (11).

### Strengths and limitations

The main strength of this systematic review is that the source studies used similar designs and had consistent definitions for exposures and outcomes. This allowed us to conduct a meta-analysis on our 10 Tier 1 studies and report an overall pooled adjusted effect measure across 3 prevalent diseases that quantifies the effectiveness of outpatient follow-up visits in reducing readmissions. Our subgroup analyses identified that study quality, disease condition, and time-dependent bias contributed to between-study heterogeneity, which illustrates the clinical complexity of addressing readmissions and highlights that the effectiveness of outpatient follow-up visits is likely affected by a myriad of patient, clinical, and system-level factors.

Our findings have some limitations. Our analysis was limited to adult patients discharged from a US hospital with heart failure, COPD, AMI, or stroke. We focused on outpatient follow-up visits that occurred in typical ambulatory settings with a physician or nurse practitioner. We excluded outpatient follow-up visits that used a designated outpatient follow-up clinic because these require organizational and financial resources beyond what is typically available to most hospitals. However, we found only 3 studies that used a dedicated outpatient follow-up clinic (46–48), all of which were conducted at a single center and had small sample sizes. Individual studies used either ORs or HRs as effect estimates, but we chose not to convert ORs to relative risks because of the limitations of the proposed methods (49–52). Individual studies varied in their range of discharge destinations, in their geographical locations (within the US), and in demographic characteristics. All these factors likely limit the generalizability of our findings. In light of these limitations, we emphasize the need for more high-quality studies that control for time-dependent bias to further elucidate the individual effect of outpatient follow-up visits on reducing 30-day all-cause readmissions.

### Conclusion

Across multiple diseases, preventing readmissions can improve the quality of life of patients and reduce illness, death, and costs (1,3–5). At a system level, reducing readmissions could increase funding to public hospitals that have received a disproportionate level of penalties from HRRP (53,54). We identified the effectiveness of outpatient follow-up visits in reducing 30-day all-cause readmissions for US patients discharged with heart failure and stroke, but found insufficient data on outpatient follow-up visits for AMI patients. Although our findings do not support the use of outpatient follow-up visits among COPD patients, these results may be related to the design and quality of these studies rather than the disease itself.
